# ESC 2019 guidelines for the diagnosis and management of chronic coronary syndromes: Recommendations for cardiovascular imaging

**DOI:** 10.1007/s00059-020-04935-x

**Published:** 2020-05-19

**Authors:** Antti Saraste, Juhani Knuuti

**Affiliations:** 1 Turku PET Centre, Turku University Hospital and University of Turku, Kiinamllynkatu 4–8, 20520 Turku, Finland; 2 Heart Center, Turku University Hospital, Turku, Finland

**Keywords:** Coronary computed tomography angiography, Echocardiography, Single-photon emission computed tomography, Cardiac magnetic resonance, Positron emission computed tomography, Koronare Computertomographie-Angiographie, Echokardiographie, Einzelphotonenemissionscomputertomographie, Kardiale Magnetresonanztomographie, Positronenemissionscomputertomographie

## Abstract

The European Society of Cardiology (ESC) has recently published new guidelines on the diagnosis and management of chronic coronary syndromes (CCS). Due to variable symptoms, objective tests are often necessary to confirm the diagnosis, exclude alternative diagnoses, and assess the severity of underlying disease. This review provides a summary of the main diagnostic strategies listed in the guidelines for evaluation of patients suspected of having obstructive coronary artery disease (CAD). Based on data from contemporary cohorts of patients referred for diagnostic testing, the pre-test probabilities of obstructive CAD based on age, sex, and symptoms have been adjusted substantially downward compared with the previous guidelines. Further, a new concept of “clinical likelihood of CAD” was introduced accounting for the impact of various risk factors and modifiers on the pre-test probability. Noninvasive functional imaging for myocardial ischemia, coronary computed tomography angiography, or invasive coronary angiography combined with functional evaluation is recommended as the initial strategy to diagnose CAD in symptomatic patients, unless obstructive CAD can be excluded by clinical assessment alone. When available, imaging tests are recommended as noninvasive modalities instead of exercise electrocardiograms.

## Abstract (de)

Die European Society of Cardiology (ESC) hat kürzlich neue Leitlinien zur Diagnose und Therapie chronischer Koronarsyndrome (CCS) publiziert. Aufgrund der Variabilität der Symptome sind häufig objektive Untersuchungen notwendig, um die Diagnose zu bestätigen, alternative Diagnosen auszuschließen und die Schwere der zugrunde liegenden Erkrankung zu bestimmen. Die vorliegende Übersichtsarbeit bietet eine Zusammenfassung der wesentlichen diagnostischen Strategien, die in den Leitlinien zur Beurteilung von Patienten mit Verdacht auf das Vorliegen einer stenosierenden koronaren Herzkrankheit (KHK) aufgeführt sind. Auf der Grundlage von Daten aktueller Patientenkohorten, die zur diagnostischen Abklärung überwiesen worden waren, wurden die Vortestwahrscheinlichkeiten einer stenosierenden KHK auf der Basis von Alter, Geschlecht und Symptomen im Vergleich zu früheren Leitlinien deutlich nach unten korrigiert. Darüber hinaus wurde ein neues Konzept der „klinischen Wahrscheinlichkeit einer KHK“ eingeführt, welches den Einfluss verschiedener Risikofaktoren und Modifikatoren der Vortestwahrscheinlichkeit berücksichtigt. Als initiale Strategie zur Diagnose einer KHK bei Patienten mit Symptomen wird die nichtinvasive funktionelle bildgebende Untersuchung auf Myokardischämie, die Computertomographie-Angiographie der Koronarien oder die invasive Koronarangiographie in Kombination mit einer Funktionsuntersuchung empfohlen, wenn die stenosierende KHK nicht durch alleinige klinische Untersuchung ausgeschlossen werden kann. Wenn verfügbar, werden bildgebende Untersuchungen als nichtinvasive Modalitäten anstelle des Belastungs-EKG empfohlen.

## Background

The European Society of Cardiology (ESC) recently published the 2019 ESC guidelines on the diagnosis and management of chronic coronary syndromes (CCS; [1]). Due to variable and often atypical symptoms, objective tests are often necessary to confirm the diagnosis of obstructive coronary artery disease (CAD), exclude alternative diagnoses, and assess the severity of underlying disease. In this article, we summarize the recommendations for cardiovascular imaging in the assessment of patients with suspected obstructive CAD in the 2019 ESC guidelines together with recent data underlying the recommendations.

The 2019 guidelines focus on the spectrum of CCS, excluding the situations in which an acute coronary event, often with coronary thrombus formation, dominates the clinical presentation [1]. The term “chronic coronary syndromes” emphasizes the fact that despite stable symptoms, CAD is a dynamic process of atherosclerotic plaque accumulation and functional alterations of coronary circulation that can be modified by lifestyle, pharmacological therapies, and revascularization, which may result in disease stabilization or regression [1]. The guidelines identified six clinical scenarios most frequently encountered in clinical practice. These included suspected CAD and “stable” angina symptoms and/or dyspnea; new onset of heart failure or left ventricular (LV) dysfunction and suspected CAD; stabilized symptoms <1 year after an acute coronary syndrome event or revascularization; asymptomatic and symptomatic patients >1 year after initial diagnosis or revascularization; angina and suspected vasospastic or microvascular disease; as well as asymptomatic subjects in whom CAD is detected at screening.

## Diagnostic approach

The diagnostic approach in a patient with suspected obstructive CAD can be described as a series of successive steps [1]. An initial step is to assess the symptoms and signs so as to exclude patients with possible unstable angina or other forms of acute coronary syndrome. In other patients, step 2 is to evaluate the patient’s general condition and quality of life. Comorbidities and other possible causes of the symptoms that potentially influence therapeutic decisions are considered. Step 3 includes basic testing and assessment of LV function. A resting transthoracic echocardiogram is recommended for all patients to exclude alternative causes of angina, identify regional wall-motion abnormalities suggestive of CAD, determine LV ejection fraction for risk-stratification purposes, as well as to evaluate diastolic function (Fig. 1; [1]). Cardiac magnetic resonance (CMR) imaging may be considered in patients with an inconclusive echocardiogram.

**Fig. 1:**
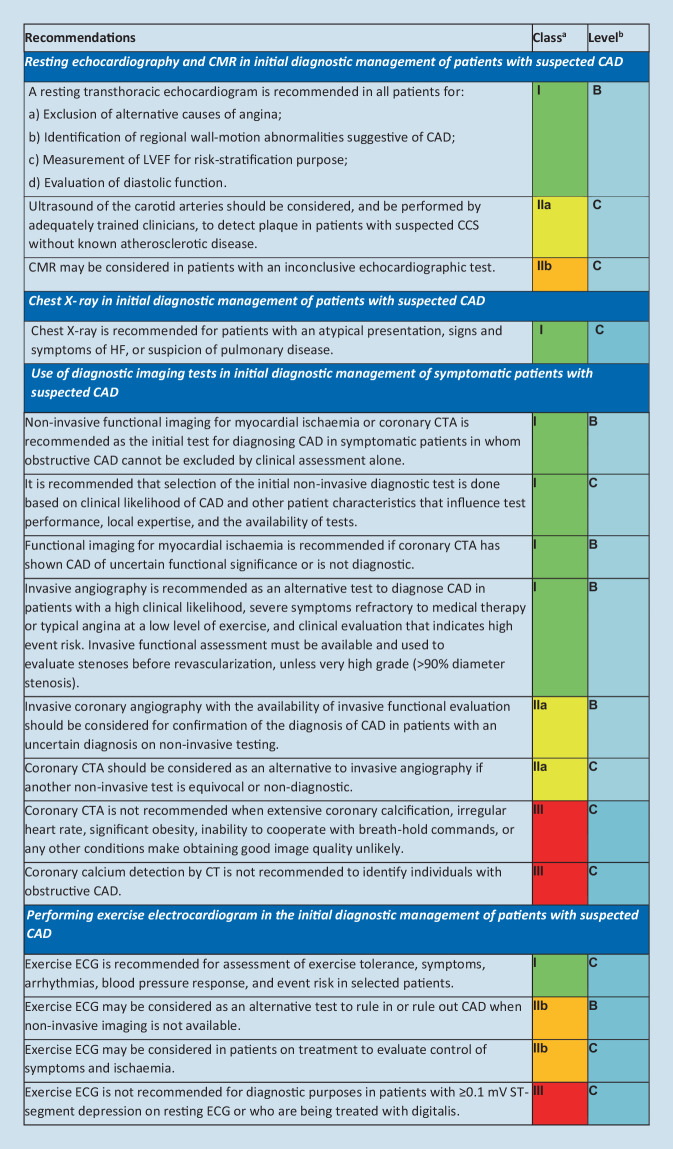
Summary of recommendations for imaging in the 2019 ESC guidelines on the management of chronic coronary syndromes [1]

**Fig. 1:**
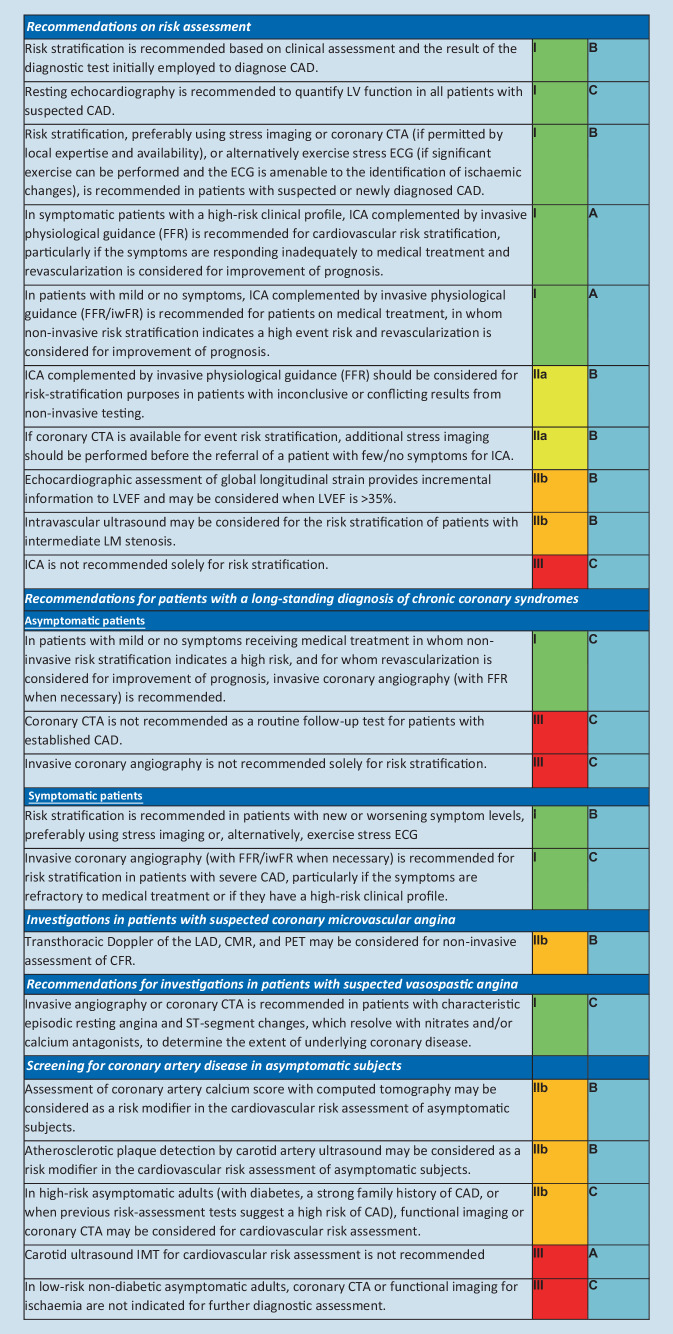
(continued) [1]

In step 4, the pre-test probability (PTP) and clinical likelihood of obstructive CAD are estimated and, on this basis, diagnostic testing strategies, either noninvasive or invasive, are offered to selected patients in order to establish the diagnosis of CAD (step 5).

Once the diagnosis of obstructive CAD has been confirmed, the patient’s event risk will be determined (step 6). Risk stratification has a major impact on therapeutic decisions, particularly identification of patients at high event risk who may benefit from revascularization beyond the amelioration of symptoms [1, 2]. Event risk stratification is recommended based on clinical assessment and the results of the diagnostic test initially employed for making the diagnosis of CAD (Fig. 1). An important part of the risk assessment is evaluation of LV ejection fraction by echocardiography. Systolic function can be reduced without a decrease in ejection fraction, and a decreased global longitudinal strain (GLS) by >2 standard deviations from the lower normal reference value has demonstrated incremental value in risk assessment of patients with CCS, especially in those with LV ejection fraction >35% [3–5].

## PTP and clinical likelihood of CAD

**Fig. 2:**
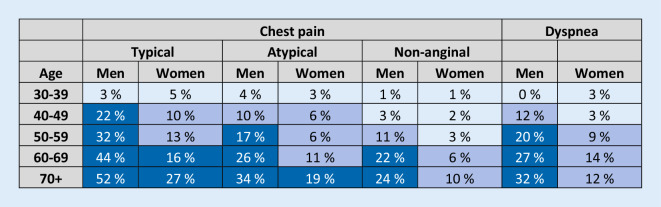
Pre-test probabilities of obstructive coronary artery disease (*CAD*) in 15,815 symptomatic patients according to age, sex, and the nature of symptoms in pooled analysis of contemporary data. In addition to the classic Diamond and Forrester classes, patients with dyspnea only or dyspnea as the primary symptom are included. The *dark blue shaded* regions denote the groups in which noninvasive testing is most beneficial (pre-test probability [PTP] >15%). The *light blue shaded* regions denote the groups with PTP of CAD between 5 and 15% in whom diagnostic testing may be considered after assessing the overall clinical likelihood based on modifiers of PTP. (Reprinted by permission of Oxford University Press on behalf of the European Society of Cardiology from reference [6])

Estimation of the PTP and clinical likelihood of obstructive CAD serves to identify patients who require further investigation or treatment (Fig. 1; [1]). The prevalence of obstructive CAD among patients with suspected CAD has substantially decreased over time. A pooled analysis [6] of three contemporary study cohorts including patients evaluated for suspected CAD [7–9] indicates that the PTP based on age, sex, and nature of symptoms in current patients is approximately one third of that predicted by the model used in the 2013 version of the ESC guidelines [10, 11]. The updated PTPs, including PTPs in patients presenting with dyspnea, are displayed in Fig. 2. It should, however, be noted that the PTPs are based mainly on patients from countries with low cardiovascular disease risk and PTP may vary between different regions and countries.

The reduced PTP has important consequences on the evaluation of patients with suspected obstructive CAD. An overestimation of PTP is probably an important contributory factor to a low diagnostic yield of noninvasive and invasive testing. The noninvasive diagnostic tests of obstructive CAD have the best performance in patients with an intermediate likelihood of disease (>15%). However, if diagnostic testing was deferred in patients with new PTP <15%, this would result in a large increase in the proportion of patients in whom diagnostic testing is not recommended. In data derived from the PROMISE (Prospective Multicenter Imaging Study for Evaluation of Chest Pain) trial, 50% of patients previously classified as having an intermediate PTP of obstructive CAD were reclassified to a PTP <15% according to the new PTP [8]. In data derived from the pooled analysis [6], 57% of all patients had a PTP <15%.

Studies have shown that an outcome in patients with the new PTP of up to 15% is good (annual risk of cardiovascular death or myocardial infarction [MI] <1%; [8, 9]). Hence, it would be safe to defer routine testing in patients with PTP <15%, thus reducing unnecessary procedures. However, currently there are no randomized controlled trials that include evaluation of outcomes with a “no-test” strategy. Therefore, performing diagnostic testing also in patients with a new PTP of 5–15% more closely reflects current clinical practice and may be considered appropriate, particularly if symptoms are limiting and require clarification [1, 12]. Patient preference, local resources and availability of tests, clinical judgment, and appropriate patient information remain important for the decision to proceed with noninvasive diagnostic testing in an individual patient when the PTP is 5–15%, and the higher likelihood of a false-positive test must be considered [1]. Patients with a very low PTP (≤5%) can be assumed to have such a low probability of disease that diagnostic testing should be performed only for compelling reasons [1].

A concept of clinical likelihood of obstructive CAD has been introduced to consider modifiers of PTP beyond age, sex, and nature of symptoms. Clinical models that incorporate information on risk factors for cardiovascular disease, resting electrocardiogram (ECG) changes (Q-wave and changes in ST-segment or T‑wave), LV dysfunction suggestive of CAD, findings of exercise ECG or coronary calcification provide improved identification of patients with obstructive CAD compared with age, sex, and symptoms alone [13–16]. The absence of coronary calcium (Agatston score = 0) is associated with a low prevalence of obstructive CAD (<5%) and a low risk of death or non-fatal MI (<1% annual risk; [17, 18]). However, coronary calcium detection by computed tomography is not recommended for identifying individuals with obstructive CAD [1]. Although the optimal use of these factors in improving the PTP assessment has not yet been established, they have implications particularly in refining the likelihood of CAD patients with PTP of 5–15% based on age, sex, and nature of symptoms.

## Diagnostic tests

Over the past few years, numerous studies have evaluated the performance of diagnostic tests and clinical trials have compared the effects of diagnostic strategies on management and clinical outcomes in patients with suspected CCS. A summary of the performance of diagnostic tests for the detection of anatomically significant (>50% stenosis) or functionally significant (fractional flow reserve, FFR ≤0.80) CAD based on recent meta-analyses is shown in Table 1 [19]. Of note, the performance of a given test in different studies varies for numerous reasons, including selection and referral bias. Therefore, differences between individual diagnostic tests as well as summary estimates based on these meta-analyses should be interpreted cautiously.

**Table 1:** Performance of diagnostic tests for the detection of anatomically significant (>50% stenosis) or functionally significant (FFR ≤0.80) CAD^a^

Test	Sensitivity (%, 95% CI)	Specificity (%, 95% CI)	+LR	−LR
*Anatomically significant CAD*				
Exercise ECG	58 (46–69)	62 (54–69)	1.53 (1.21–1.94)	0.68 (0.49–0.93)
Stress echo	85 (80–89)	82 (72–89)	4.67 (2.95–7.41)	0.18 (0.13–0.25)
Coronary CTA^b^	96 (93–98)	82 (75–87)	8.9 (6.1–13.5)	0.022 (0.01–0.04)
SPECT	87 (83–90)	70 (63–76)	2.88 (2.33–3.56)	0.19 (0.15–0.24)
PET	90 (78–96)	85 (78–90)	5.87 (3.40–10.15)	0.12 (0.05–0.29)
Stress CMR	90 (83–94)	80 (69–88)	4.54 (2.37–8.72)	0.13 (0.07–0.24)
*Functionally significant CAD*				
Coronary CTA	93 (89–96)	53 (37–68)	1.97 (1.28–3.03)	0.13 (0.06–0.25)
SPECT	73 (62–82)	83 (71–90)	4.21 (2.62–6.76)	0.33 (0.24–0.46)
PET	89 (82–93)	85 (81–88)	6.04 (4.29–8.51)	0.13 (0.08–0.22)
Stress CMR	89 (85–92)	87 (83–91)	7.10 (5.07–9.95)	0.13 (0.09–0.18)

*CAD* coronary artery disease, *CTA* computed tomography angiography, *CI* confidence interval, *ECG* electrocardiogram, *FFR* fractional flow reserve, *LR* likelihood ratio, *PET* positron emission tomography, *SPECT* single-photon emission computed tomography (exercise SPECT with or without dipyridamole or adenosine), *Stress CMR* stress cardiac magnetic resonance, *Stress echo* exercise stress echocardiography, *+LR* positive likelihood ratio, *−LR* negative likelihood ratio

^a^Modified from [19]

^b^Data from [20]

### Anatomical imaging

Coronary CTA is an anatomical imaging modality that allows for visualization of the coronary artery lumen and wall using an intravenous contrast agent. Coronary CTA provides very high sensitivity for the detection of coronary artery stenoses defined as obstructive by invasive coronary angiography (ICA; Table 1; [19, 20]) as well as nonobstructive calcified or noncalcified plaques. The specificity of coronary CTA is lower than its sensitivity, particularly in studies using invasive FFR rather than ICA as the reference standard (Fig. 1). Stenoses estimated to be 50–90% by visual inspection are not necessarily functionally significant, i.e., they do not always induce myocardial ischemia. Therefore, either noninvasive or invasive functional testing is recommended for further evaluation of angiographic stenosis detected by coronary CTA or invasive angiography, unless a very high grade (>90% diameter stenosis) stenosis is detected via invasive angiography [1].

Poor image quality and severe calcifications may lead to overestimation of stenosis severity by coronary CTA, particularly by non-experienced readers [21]. Therefore, coronary CTA is not recommended when extensive coronary calcification, irregular heart rate, significant obesity, inability to cooperate with breath hold commands, or any other condition makes good image quality unlikely [1]. In patients with previous revascularization (bypass grafts, stents), the accuracy of coronary CTA is frequently impaired by blooming artifacts and incomplete evaluation of native vessels [22]. Acquisition protocols for coronary CTA should include special measures to keep radiation exposure as low as possible [23].

Prospective registries have shown that the absence of stenosis in coronary CTA is associated with good prognosis [24, 25]. The PROMISE trial randomized 10,003 symptomatic patients referred to noninvasive testing for suspected CAD to either coronary CTA or functional testing as the first diagnostic test [26]. The trial demonstrated no difference in the primary outcome of all-cause mortality, myocardial infarction, hospitalization for unstable angina, or major complications of cardiovascular procedures or diagnostic testing between coronary CTA and functional testing after 25 months of follow-up (3.3% vs. 3.0%; [26]). The randomized SCOT-HEART (Scottish Computed Tomography of the Heart) trial included 8000 patients with suspected obstructive CAD and demonstrated significantly lower rate of the combined endpoint of cardiovascular death or nonfatal MI (2.3% vs. 3.9% during 5‑year follow-up) in patients for whom coronary CTA was performed in addition to routine testing, which consisted predominantly of exercise ECG [27, 28]. Rates of ICA and revascularizations were not different among the groups, but preventive therapies were more often initiated in the CTA group than in the control group [28].

Coronary CTA can be complemented by stress computed tomography (CT) myocardial perfusion imaging or off-line “virtual” CT-based FFR (FFRCT) using datasets acquired by CT at rest to improve detection of functionally significant CAD defined by invasive FFR [29]. Results of retrospective registries, trial substudies, and small randomized trials have demonstrated that non-ischemic FFRCT results are associated with a favorable prognosis [30–32]. In the absence of comparative prospective outcome trials, these emerging modalities were discussed in the guidelines, but no specific recommendations on their use were given.

### Functional imaging tests

**Fig. 3:**
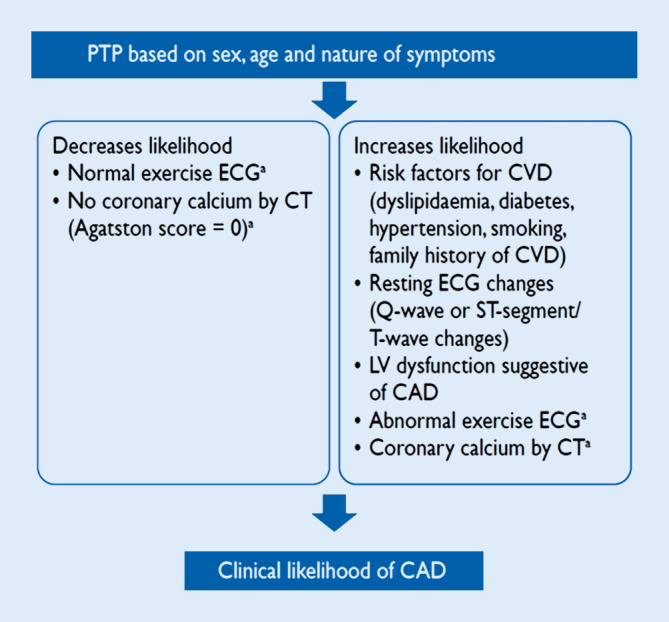
Determinants of clinical likelihood of obstructive CAD. *CAD* coronary artery disease, *CT* computed tomography, *CVD* cardiovascular disease, *ECG* electrocardiogram, *LV* left ventricular, *PTP* pre-test probability. ^a^When available. (Reprinted by permission of Oxford University Press on behalf of the European Society of Cardiology, www.escardio.org/Guidelines, from [1])

Functional imaging for ischemia includes myocardial perfusion imaging with single-photon emission computed tomography (SPECT) or positron emission computed tomography (PET), stress echocardiography, or stress CMR. Detection of obstructive CAD is based on perfusion abnormalities or ischemic wall motion abnormalities provoked by exercise or pharmacological stress (Fig. 3).

Compared with coronary CTA, functional imaging tests have higher specificity for the detection of hemodynamically significant coronary stenosis in studies that have used invasive FFR as the reference standard (Table 1; [19]). Ischemia on noninvasive testing has also been shown to predict symptom relief upon revascularization [33]. In comparative trials and a network meta-analysis, the use of functional imaging tests resulted in fewer referrals for ICA compared with a strategy relying on anatomical imaging or exercise ECG [34–37]. It should, however, be noted that the presence of coronary atherosclerosis that does not cause luminal narrowing to the extent that provokes myocardial ischemia remains undetected by functional testing [38]. Therefore, in the presence of a negative functional test result, patients should receive risk-factor modification based on commonly applied risk charts and recommendations [39].

Functional imaging tests are effective diagnostic tools for risk stratification of patients with CCS. A normal functional test result is associated with a low (≤1% per year) subsequent rate of cardiac death and MI [40]. By contrast, stress-induced wall motion abnormalities or reversible perfusion defects corresponding to ≥10% of the total LV myocardium have been reported across a number of prognostic series to denote moderate–severe ischemia associated with a high event rate in CCS (annual rate of cardiovascular death or MI >3%; [41]). Based on observational studies, these patients may benefit from ICA and revascularization [42]. However, after publication of the guidelines, the prospective randomized ISCHEMIA (International Study of Comparative Health Effectiveness with Medical and Invasive Approaches) trial did not find evidence that an initial invasive strategy, as compared with an initial conservative strategy, reduced the risk of ischemic cardiovascular events or death from any cause over a median of 3.2 years in such patients [43].

## Role of exercise electrocardiogram

Compared with exercise ECG, noninvasive functional imaging tests not only have the advantage of indicating the location of ischemia, but also of superior diagnostic performance for the detection of obstructive CAD (Table 1; [19]). Exercise ECG has limited power to rule in or rule out obstructive CAD (Table 1; [19]). As discussed earlier, randomized clinical trials have shown that the addition of coronary CTA or functional imaging clarifies the diagnosis, enables targeting of preventive therapies and interventions, and potentially reduces the risk of MI compared with a diagnostic workup relying on exercise ECG. Therefore, the 2019 ESC guidelines recommend the use of an imaging diagnostic test instead of exercise ECG as the initial noninvasive test for diagnosing obstructive CAD always if possible [1]. However, an exercise ECG provides complementary clinically useful information beyond ECG changes and valuable prognostic information. Therefore, exercise ECG has a role in the assessment of symptoms, ST-segment changes, exercise tolerance, arrhythmias, blood pressure response, and event risk [1], and thereby helps to inform about the clinical likelihood of CAD as well as an appropriate diagnostic and therapeutic strategy.

## Selection of appropriate testing

**Fig. 4:**
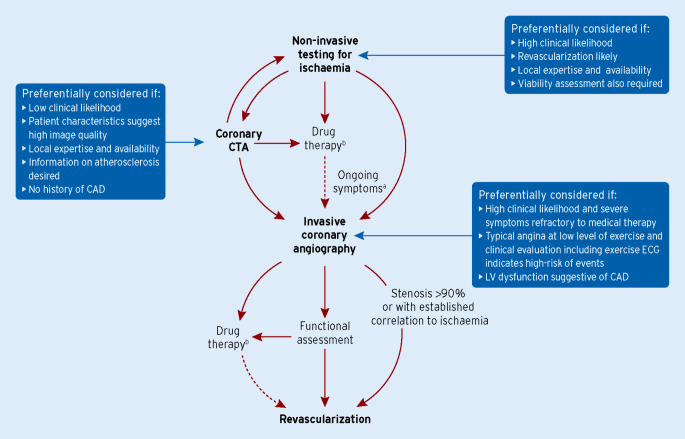
Main diagnostic pathways in symptomatic patients with suspected obstructive coronary artery disease. *CAD* coronary artery disease, *CTA* computed tomography angiography, *ECG* electrocardiogram, *LV* left ventricular. ^a^Consider microvascular angina. ^b^Antianginal medications and risk factor modification. (Reprinted by permission of Oxford University Press on behalf of the European Society of Cardiology, www.escardio.org/Guidelines, from [1])

The main diagnostic pathways in symptomatic patients with suspected obstructive CAD according to the 2019 ESC guidelines are summarized in Fig. 4 [1]. Depending on clinical conditions and the healthcare environment, patient workup can start with noninvasive functional testing, coronary CTA, or ICA. Through each pathway, both functional and anatomical information is gathered to inform an appropriate diagnostic and therapeutic strategy. Risk-factor modification should be considered for all patients.

Either functional imaging for myocardial ischemia or coronary CTA is recommended as the initial noninvasive tests for diagnosing CAD in symptomatic patients in whom obstructive CAD cannot be excluded by clinical assessment alone [1]. It is recommended that the choice of the initial noninvasive diagnostic test is based on the clinical likelihood of CAD and other patient characteristics that influence test performance as well as on local expertise and availability of tests.

**Fig. 5:**
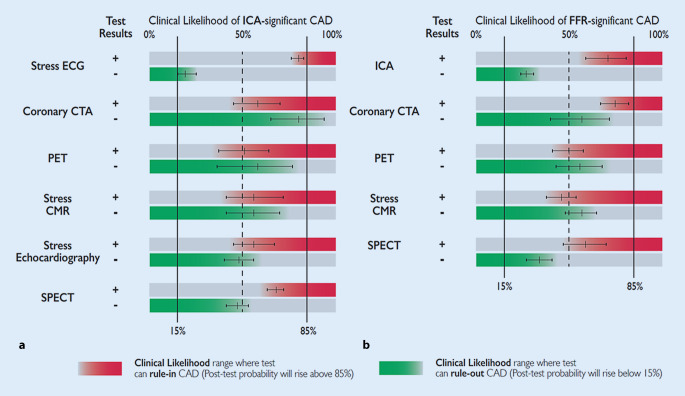
Ranges of clinical likelihood of CAD in which a given test can rule in (*red*) or rule out (*green*) obstructive CAD. The graph displays in *red* the range of clinical likelihood of CAD when a given test can rule in CAD when positive. The *green* part shows the range of clinical likelihood of CAD when a given test can rule out CAD when negative. The ideal range of clinical likelihood for a given test is when the red and green colors overlap and the test can simultaneously rule in and rule out CAD depending the test result. **a** Reference standard is anatomical assessment using ICA. **b** Reference standard is functional assessment using FFR. Note, in **b**, the data with stress echocardiography and SPECT are more limited than with the other techniques. The *crosshairs* mark the mean value and their 95% confidence intervals. *CAD* coronary artery disease, *CMR* cardiac magnetic resonance, *CTA* computed tomography angiography, *ECG* electrocardiogram, *FFR* fractional flow reserve, *ICA* invasive coronary angiography, *PET* positron emission tomography, *SPECT* single-photon emission computed tomography. (Reprinted by permission of Oxford University Press on behalf of the European Society of Cardiology, www.escardio.org/Guidelines, from [1])

The likelihood ratios of diagnostic tests constitute useful parameters of their ability to correctly classify patients, and can be used to facilitate the selection of the most useful test in any given patient [19]. Each noninvasive diagnostic test has a particular range of clinical likelihood of obstructive CAD where the usefulness of its application is maximal. Given a clinical likelihood of obstructive CAD and the likelihood ratios of a particular test, one can assess the post-test probability of obstructive CAD after performing such a test. Using this approach, one can estimate the optimal ranges of clinical likelihood for each test where they can reclassify patients from intermediate to either low or high post-test probability of CAD (Fig. 5; [19]).

Coronary CTA is the preferred test in patients within the lower range of clinical likelihood of CAD, no previous diagnosis of CAD, and characteristics associated with a high likelihood of good image quality. It can accurately rule out both anatomically and functionally significant CAD, and detect subclinical coronary atherosclerosis (Fig. 5). Coronary CTA should also be considered as an alternative to ICA if another noninvasive test is equivocal or nondiagnostic.

The noninvasive functional tests for ischemia typically have better rule-in power. For revascularization decisions, functional evaluation of ischemia (either noninvasive or invasive) is required in most patients. Therefore, functional noninvasive testing may be preferred in patients at the higher range of clinical likelihood, if revascularization is likely or the patient has previously been diagnosed with CAD. Functional imaging for myocardial ischemia is also recommended if coronary CTA has shown CAD of uncertain functional significance or is not diagnostic.

Direct ICA is recommended as an alternative to noninvasive testing in order to diagnose CAD in patients with a high clinical likelihood and severe symptoms refractory to medical therapy or typical angina at a low level of exercise and clinical evaluation that indicates high event risk [1]. Invasive functional assessment must be available and used to evaluate stenoses before revascularization, unless they are of very high grade (>90% diameter stenosis).

Risks related to different diagnostic tests need to be weighed against the benefits to the individual [44]. For example, exposure to ionizing radiation associated with coronary CTA and nuclear perfusion imaging needs to be taken into account, especially in young individuals. Similarly, contraindications to pharmacological stressors and contrast agents (iodine-based contrast agents and gadolinium-based chelates) need to be considered. When testing is used appropriately, the clinical benefit from accurate diagnosis and therapy exceeds the projected risks of testing itself [44].

## Angina without obstructive disease in the epicardial coronary arteries

The possibility of a microcirculatory origin of angina should be considered in patients with clear-cut angina and coronary vessels that are either normal or have mild stenosis deemed functionally nonsignificant on ICA or CTA. Impaired microcirculatory conductance can be diagnosed by measuring coronary flow reserve noninvasively with transthoracic Doppler echocardiography (by imaging left anterior descending flow; [45]), CMR (myocardial perfusion index; [46]), or PET [47]. However, noninvasive methods provide limited assessment of microvascular function, because assessment of endothelial function (arteriolar dysregulation) in the coronary microcirculation requires selective acetylcholine infusion into the epicardial vessels. In patients with suspected vasospastic angina and documented ECG changes, coronary CTA or ICA is indicated to rule out the presence of fixed coronary stenosis.

## Conclusion

The pre-test probability (PTP) estimations of coronary artery disease (CAD) based on age, sex, and nature of symptoms have undergone a major update with significantly lower probabilities than in previous estimates. In addition, a new phrase—“clinical likelihood of CAD”—that incorporates other modifiers of PTP beyond age, gender, and nature of symptoms has been introduced. Noninvasive functional imaging for myocardial ischemia, coronary computed tomography angiography, or invasive coronary angiography combined with functional evaluation may be used as the initial test to rule out or establish the diagnosis of chronic coronary syndromes. Selection of the initial noninvasive diagnostic test is based on the clinical likelihood of CAD, the test performance in ruling in or ruling out obstructive CAD, patient characteristics, local expertise, and the availability of the test. For revascularization decisions, both anatomy and functional evaluation are to be considered.
